# Ultrasound assessment of haemoperitoneum in ectopic pregnancy: derivation of a prediction model

**DOI:** 10.1186/1749-7922-2-23

**Published:** 2007-09-07

**Authors:** Arnaud Fauconnier, Ali Mabrouk, Laurent J Salomon, Jean-Pierre Bernard, Yves Ville

**Affiliations:** 1 Department of Gynaecology, Obstetrics and Reproductive medicine, CHI Poissy-St-Germain, Saint-Germain-En-Laye, France

## Abstract

**Background:**

To derive an ultrasound-based prediction model for the quantification of haemoperitoneum in ectopic pregnancy (EP).

**Methods:**

Retrospective study of 89 patients operated upon EP between January 1999 and March 2003 in a French Gynaecology and Obstetrics department in a university hospital. Transvaginal sonograms, clinical and biological variables from patients with haemoperitoneum ≥ 300 ml at surgery were compared with those from patients with haemoperitoneum < 300 ml or no haemoperitoneum. Sensitivity, specificity, positive and negative likelihood ratios were calculated for each parameter after appropriate dichotomization. Multiple logistic regression analysis was used to select the best combination at predicting haemoperitoneum ≥ 300 ml.

**Results:**

Three parameters predicted haemoperitoneum ≥ 300 ml independently:  moderate to severe spontaneous pelvic pain, fluid above the uterine fundus  or around the ovary at transvaginal ultrasound, and serum haemoglobin concentration < 10 g/dL. A woman with none of these three criteria would have a probability of 5.3% for haemoperitoneum ≥ 300 ml. When two or more criterias were present, the probability for haemoperitoneum ≥ 300 ml reached 92.6%.

**Conclusion:**

The proposed model accurately predicted significant haemoperitoneum in patients diagnosed to have EP.

## Background

The combination of transvaginal ultrasound (US) and serum human chorionic gonadotropin (hCG) determination has proven to be reliable for the early diagnosis of ectopic pregnancy (EP) [1-4]. The goal of early diagnosis is the prevention of tubal rupture [5], which is the cause of most EP-related deaths [6,7], and is a surgical emergency. Accordingly, suspicion of tubal rupture is an absolute contraindication for medical treatment of EP using methotrexate [8-12].

Although, the diagnosis of tubal rupture is obvious when patients are haemodynamically unstable, symptoms in most cases of tubal rupture are more subtle [13-16]. The only diagnostic method to rule out tubal rupture would be to perform a laparoscopy in all cases [15].

However, considering that most tubal rupture would cause significant haemoperitoneum and that the latter would often correspond to tubal rupture is clinically sound. Evidence for haemoperitoneum is usually found on US examination. Nevertheless, the various US criteria used to quantify the amount of the haemoperitoneum prior to surgery [12,17-21] have not been validated yet.

The aim of our study was therefore to identify criterias in the pre operative work-up that could be useful to predict the haemoperitoneum volume, in a homogeneous series of patients operated upon EP.

## Methods

### Study population

All patients assigned for surgical treatment for EP between January 1999 and March 2003, by laparoscopy or by laparotomy, were included in the study.

The patients were identified from the hospital's computerized Medical Information System. The indication for surgical treatment of EP was left to the attending consultant gynaecologist, and surgery took place either at the time of presentation or after failure of medical treatment using methotrexate. At our institution medical treatment is offered on the basis of: absence of significant pain and stable hemodynamics, normal serum haemoglobin concentration and white cell count as well as serum hCG concentration < 5000 IU/L; patient accepting the medical treatment and its follow-up.

### Variables of interest

The following parameters were collected from the medical records: age, gravidity, parity, gestational age, the existence of vaginal bleeding, the existence and severity of spontaneous pelvic pain, systolic and diastolic blood pressure, pulse rate, pre operative serum haemoglobin concentration (g/dL) and pre operative serum hCG concentration (UI/L). All these parameters were collected at presentation. The severity of spontaneous pelvic pain was subjectively assessed by the specialist registrar, in a semi-quantitative fashion using four levels: absent, mild, moderate, or severe [22].

Transvaginal US investigation was carried out pre operatively in all cases by the registrar or the consultant. One of three US machines were used: a Voluson^® ^530D MT, fitted with a 7.5 MHZ convex transvaginal probe S-EW5/7K (Kretztechnik AG), a Logic 700, fitted with a 5.6 MHZ convex transvaginal probe 618E (GE Medical System), and a Logic 500 fitted with a 4.8 MHZ convex transvaginal probe E721 (GE Medical System). It is the policy in our institution to record images of a standardised sonogram in cases with EP. At least, three pictures are requested: one of the uterus in a stricly midsagittal plane from the uterine cervix to the fundus; one of each ovary in there greater diameter. In case of abnormal extra-uterine finding one or more picture were requested. All sonograms were carefully reviewed for the study, in order to ensure a standardised analysis of the characteristics of intraperitoneal effusion. All ultrasound records were reviewed by one trained specialist registrar (AM) under the direct supervision of a senior operator skilled in the practice of gynaecological ultrasound (J-PB). This review was carried out blindly to the quantification of haemoperitoneum as stated in the surgery report. The presence of free fluid in the Pouch of Douglas was defined by the presence of liquid behind the uterus on a strictly midsagittal plane. The volume of the intraperitoneal fluid was assessed in semi-quantitative way according to the level reached relative to the uterus on a midsagittal plane: (i) below or at the level of the uterine isthmus; (ii) reaching the uterine body; (iii) exceeding the uterine fundus. This classification was adapted from another study [18]. Fluid around the ovary or in the vesico-uterine pouch was also noted. The fluid was described as echogenous if fine, diffuse echos were visible in the liquid [23,24], and the presence of clots was noted if there were heterogeneous areas within the fluid [25].

A quantitative estimate of the haemoperitoneum in ml, was carried out during surgery by recording of the volume aspirated and irrigation fluid. Presence or absence of tubal rupture was also noted.

### Statistical analysis

The study population was split into two groups according to the volume of the haemoperitoneum found at surgery: one group of patients with a haemoperitoneum volume quantified equal to or above the median volume; one group of patients with no haemoperitoneum or below the median volume.

The two groups were compared using Pearson's Chi-Square test for qualitative variables and Student's t test for quantitative variables. The continuous and ordinal variables found to be associated with haemoperitoneum equal to or above the median volume at a threshold of p < 0.10 in the univariate analysis were then dichotomised to enable their use in a prediction model. The diagnostic value of each variable was estimated by calculating the sensitivity (Se), specificity (Sp), positive likelihood ratio (LR) and negative LR. When a variable presented several possible cut-off levels, the one chosen was either that which gave maximum Se or maximum Sp [26].

Multiple logistic regression analysis was then used in order to select the best combination of variables to predict the presence of a haemoperitoneum equal to or above the median volume. The condition for variables to be included in the logistic regression model was the fact of presenting a sufficiently high diagnostic value: negative LR ≤ 0.25 for variables with high sensitivity [26]; or positive LR ≥ 4 for variables with high specificity [26]. A backward stepwise procedure was then used to remove variables so that the final model included only those variables independently associated with haemoperitoneum equal to or above the median volume at a threshold of p < 0.05.

The variables thus selected were then combined with each other and the diagnostic performance of each of the various combinations for prediction of haemoperitoneum equal to or above the median volume was assessed by calculating Se, Sp, positive and negative LR on the basis of the patients in our sample population.

Analyses were carried out using the version 5.0 Statview statistics software package (SAS Corporation).

## Results

During the study period 119 patients were operated upon EP, of whom 30 (25.2%) were excluded, as no adequate US image were available (N = 15) or when the haemoperitoneum had not been measured intraoperatively (N = 15). This left 89 patients to constitute the study population. Baseline characteristics of the population are shown in Table 1.

**Table 1:** Characteristics of the study population (N=89)

variables	n	Mean ± 1 SD	Frequency (%)
Age (years)		30.2 ± 5.0	
Gravidity		2.9 ± 1.6	
Gestational age (days)		32.9 ± 16.2	
Laparoscopy			
From outset	76		85.4
Methotrexate failure	11		12.4
Laparotomy	2		2.2
Tubal rupture	36		40.4
Haemoperitoneum (mL)			
<60	21		23.6
[60–300]	20		22.5
[300–900]	37		41.6
≥ 900	11		12.3

SD = standard deviation

The median volume of the haemoperitoneum measured at the time of surgery was 300 ml. Forty eight (53.9%) patients presented haemoperitoneum ≥ 300 ml and 41 patients (46.1%) presented no haemoperitoneum or < 300 ml (Table 1). Thirty eight patients had tubal rupture (40.4%). The proportion of ruptured tubes was markedly higher among patients with a haemoperitoneum ≥ 300 ml (60.4 versus 17.1%; p < 0.0001).

The crude associations between the studied variables and the existence of massive haemoperitoneum are summarised in Table 2. There was a linear trend between the volume of intraperitoneal fluid at transvaginal sonogram of the uterus and the probability of haemoperitoneum ≥ 300 ml at surgery: indeed, 83.3% of patients with fluid above the uterine fundus, 70.6 with fluid reaching the uterine body and 24.3% with fluid under the uterine isthmus or absent had haemoperitoneum ≥ 300 ml respectively (Chi-square for linear trend = 20.9 with 1 degree of freedom; p < 0.0001).

**Table 2:** Characteristics and ultrasound findings according to the haemoperitoneum volume at surgery

Variables	Haemoperitoneum<300 ml N = 41	Haemoperitoneum≥300 ml N = 48	P value
	Mean ± 1 SD	Mean ± 1 SD	
Age (year)	30.8 ± 5.5	29.7 ± 4.7	0.301*
Number of previous pregnancies	3.0 ± 1.8	2.9 ± 1.6	0.718*
Parity	1.2 ± 1.2	1.0 ± 0.9	0.400*
Gestional age (day)	34.7 ± 15.5	31.3 ± 16.9	0.336*
	No (%)	No (%)	
Vaginal bleeding	33 (80.4)	30 (62.5)	0.063*
Spontaneous pelvic pain			
Absent	7 (17.1)	0 (0)	
Mild	13 (31.7)	4 (8.3)	<0.001^†^
Moderate	12 (29.3)	19(39.6)	
Severe	9 (21.9)	25 (52.1)	
	Mean ± 1 SD	Mean ± 1 SD	
Systolic blood pressure (mmHg)	119.5 ± 11.7	113.3 ± 15.3	0.038*
Diastolic blood pressure (mmHg)	72.4 ± 8.9	66.7 ± 10.0	0.006*
Heart rate (pulse/mn)	83.8 ± 13.8	85.5 ± 12.3	0.530*
Serum hCG concentration (UI/L)	7412 ± 11667	3828 ± 6465	0.068*
Serum haemoglobin concentration (g/dl)	12.0 ± 1.2	10.5 ± 1.9	<0.001*
Volume of intraperitoneal fluid at TVUS	No (%)	No (%)	
Absent	11 (26.8)	4 (8.3%)	
≤ Uterine isthmus	17 (41.5)	5 (10.4%)	<0.001†
Uterine body	10(24.4)	24(50.0%)	
> Uterine fundus	3 (7.3)	15(31.3%)	
Clots present at TVUS	12 (29.3)	35 (72.9)	<0.001†
Fluid around the ovary at TVUS	1 (2.4)	8 (16.7)	0.027†
Fluid in the vesico-uterine pouch at TVUS	5 (12.8)	5 (10.6)	0.753†
	Mean ± 1 SD	Mean ± 1 SD	
Height of fluid in Pouch of Douglas (mm ± 1 SD)‡	20.8 ± 10.2	26.5 ± 12.0	0.039*
Width of fluid in Pouch of Douglas (mm ± 1 SD)‡	29.3 ± 25.9	50.8 ± 24.3	<0.001*

SD = standard deviation. TVUS = transvaginal ultrasound examination

* Student's t-test.

† Chi square for heterogeneity.

‡ for one woman the measurement was not available.

The diagnostic values of the variables associated with haemoperitoneum ≥ 300 ml in the univariate analysis are shown in Table 3. Three variables had a high sensitivity with sufficiently low negative LR: any pelvic pain, moderate to severe pelvic pain, and the presence of any amount of fluid in the pouch of Douglas (Table 3). Four variables a had high specificity and sufficiently high positive LR: a serum haemoglobin concentration < 10 g/dl, a systolic blood pressure < 100 mm Hg, fluid above the uterine fundus, and fluid around the ovary (Table 3). As the latter two variables were not inter-connected (Chi-square with 1 degree of freedom = 1.1; p = not significant) and each represented a small number of patients, they were combined into a single variable: fluid above the uterine fundus OR fluid around the ovary.

**Table 3:** Diagnostic value of the variables found to be predictive of haemoperitoneum ≥ 300 ml at univariate analysis at their best cut-off point

Variable	Patients with the characteristic	Se(%)	Sp(%)	LR +	LR -	p-value*
Vaginal bleeding absent	26	37.5	80.5	1.92	0.78	0.063
Any pelvic pain	82	100.0	17.1	1.21	0.00	0.003
Moderate to severe pelvic pain	65	91.5	48.8	1.8	0.2	<0.001
Systolic blood pressure < 100 mm Hg	12	20.8	95.1	4.27	0.83	0.0215
Hb < 10 g/dl	15	29.2	97.6	11.9	0.73	0.001
Serum hCG < 5000 IU/L	66	83,3	36,6	1,31	0,46	0,032
Any fluid in the pouch of Douglas at TVUS	74	91.7	26.8	1.25	0.31	0.020
Fluid > uterine isthmus at TVUS	52	81.3	68.3	2.56	0.27	<0.001
Fluid > uterine fundus at TVUS	18	31.1	92.7	4.27	0.74	0.005
Fluid arround the ovary at TVUS	9	16.7	97.6	6.83	0.85	0.027
Fluid > uterine fundus OR fluid arround the ovary at TVUS	24	43.8	92.7	5.98	0.61	<0.001
Fluid height ≥ 11 mm at TVUS	65	85.1	39.0	1.40	0.38	0.010
Fluid Width ≥ 20 mm at TVUS	66	89.4	41.5	1.53	0.26	0.001
Clots present at TVUS	47	72.9	70.7	2.5	0.4	<0.001

Hb = Serum haemoglobin concentration. LR + = positive likelihood ratio. LR - = negative likelihood ratio. Se = sensitivity. Sp = specificity. TVUS = transvaginal ultrasound examination.

* Chi square for heterogeneity.

These variables were all introduced into a multiple regression analysis model. After using the selection procedure, three variables independently predicted the finding of a haemoperitoneum ≥ 300 ml at surgery: moderate to severe pelvic pain, fluid above the uterine fundus OR fluid around the ovary, and a serum haemoglobin concentration < 10 g/dL (Table 4). Although Serum hCG < 5000 had no interesting diagnostic value (Table 3) it was still included in the multiple regression analysis model in order to avoid any confusion bias. Indeed the Serum hCG concentration was used to select patients who may undergo medical therapy.

**Table 4:** Independent predictors of haemoperitoneum ≥ 300 ml using multiple regression analysis

Variables	Adj. OR*	95% CI
Hb < 10 g/dL		
No	1	Ref.
Yes	20.0	1.9–214.1
Spontaneous pelvic pain		
None to mild	1	Ref.
Moderate to severe	11.5	2.3–57.2
Fluid > uterine fundus OR fluid around the ovary at TVUS		
No	1	Ref.
Yes	12.9	2.5–65.5

Hb = Serum haemoglobin concentration. Adj. OR = Adjusted Odds Ratio. CI = confidence interval. Ref. = reference class for the variable.

* The variable Serum hCG < 5000 was introduced in the model in order to avoid confusion bias

Assessment for the best combination of these three variables is given in Table 5. Given a 53.9% prevalence of haemoperitoneum ≥ 300 ml in the overall population, a patient with none of the three criteria would have a probability of 5.3% for a haemoperitoneum ≥ 300 ml. Inversely when two or more criteria are present, the probability for a haemoperitoneum ≥ 300 ml is in between 92.6 and 100.0%.

**Table 5:** Classification and diagnostic value of the different combinations of the independent criterias predicting haemoperitoneum ≥ 300 ml

	Patients with the characteristic	probability of haemoperitoneum ≥ 300 ml	Se (%)	Sp (%)	LR +	LR -
Overall population	89	0.54				
						
Low risk of massive haemoperitoneum						
Absent to mild pelvic pain AND Hb ≥ 10 g/dL AND no fluid above the uterus or around the ovary at TVUS	19	0.05	97.9*	43.9*	1.75*	0.05*
						
High risk of massive haemoperitoneum						
Moderate to severe pelvic pain AND Hb <10 g/dL	14	0.93	27.1	97.6	11.10	0.75
Moderate to severe pelvic pain AND fluid above the uterus or around the ovary at TVUS	20	0.95	39.6	97.6	16.23	0.62
Two criteria present †	27	0.93	52.1	95.1	10.68	0.50
All of the 3 criteria present †	7	1.00	14.6	100.0	∞	0.85

Hb = serum haemoglobin concentration. TVUS = transvaginal ultrasound examination.

* Calculated for the absence of the disease. † All women with fluid above the uterus or around the ovary at transvaginal ultrasound examination and Hb < 10 g/dL had moderate to severe pain.

**Figure 1:**
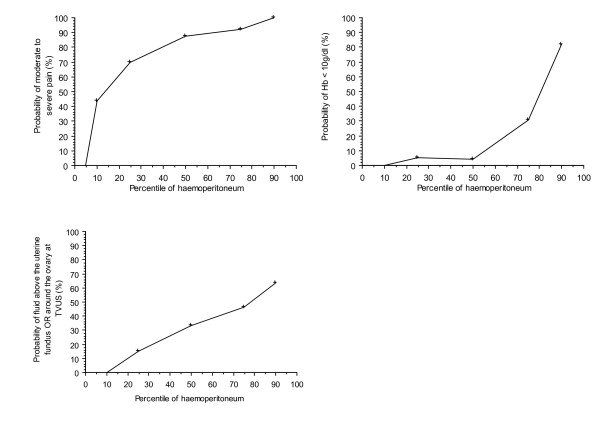
Relationship between the amount of haemoperitoneum (expressed in percentile) and the three variables used in the model.

Relations between the quantification of haemoperitoneum, expressed in percentile, and the three variables of the model are plotted on figure 1. There is an increasing relationship between the amount of the haemoperitoneum and each of the three variable used in the prediction model.

## Discussion

We have developed a clinical prediction model for the diagnosis of significant haemoperitoneum in patients with EP. This model was based on three simple and reproducible criteria: The presence of moderate to severe spontaneous pelvic pain, the presence of intraperitoneal fluid above the uterine fundus or around the ovary at transvaginal US, and the existence of a serum haemoglobin concentration < 10 g/dL. We found that this model may reliably rule out or confirm preoperatively, the existence of a significant haemoperitoneum at surgery.

One of the biggest limitations of our study is that it was retrospective. As an example, assessment of the severity of the pain was based on the attending phycisian's interpretation of the patient's pain and no strict criteria such as self-evaluation by a visual analog scale was used [27]. This may lead to a substantial interobserver difference in graded pain. There is nonetheless a fair agreement between physicians and patients' estimates of pain [28]. The quality of US data used in this study relies in the fact the fact that the information was not gathered from a written report in the patient's notes, but that they were obtained by re-interpreting standardised sonograms blind to surgical findings. Patients for whom we did not retrieve standardised transvaginal sonograms were excluded from the study. Nevertheless, prospective validation studies of samples from different centres are important to evaluate the actual predictive ability of a diagnostic model [29].

Although our criteria appeared to be reliable to exclude the presence of massive haemoperitoneum, there may be a tubal rupture in the absence of any significant haemoperitoneum in up to 27% of the cases [17,30]. However, this type of tubal rupture is probably unlikely to cause active bleeding at the time of diagnosis, but secondary resumption of bleeding could explain some of the failures observed after treatment by methotrexate, including cases with no intraperitoneal effusion [11,19]. This was the case for two patients in our study who had a tubal rupture with no active bleeding and a haemoperitoneum of 30 ml and 120 ml respectively. Anyway, our model, which was specifically designed to predict the existence of massive haemoperitoneum, is in agreement with a previously developed model for prediction of tubal rupture or active bleeding in EP [15]. Indeed, in this model, the three most important criteria were the existence of an intraperitoneal effusion at transvaginal US, any spontaneous abdominal pain and Hb ≤ 9.7 g/dl. The match between this model and ours confirms the fact that pre operative diagnosis of tubal rupture is mostly based on the presence of haemoperitoneum.

Appropriate quantification of intraperitoneal fluid at US is important because fluid in the Pouch of Douglas may be present in up to 80% of EP whether ruptured or not [17,31]. A semi-quantitative estimate of haemoperitoeum volume, using transvaginal US examination of the uterus in a midsagittal plane, which we have used (adapted from [18]) showed an excellent correlation with the volume of haemoperitoneum measured during surgery. We elected to use this classification because it is based on a standard ultrasound plane that is easy to obtain, and on criteria that seem reproducible. Two other ultrasound classifications have been proposed to assess the quantity of haemoperitoneum. One author [20] differentiates three classes of effusion: ≤ 10 ml, 10 ml to 100 ml and > 100 ml, without however setting any precise ultrasound criteria. Others [21,32] subjectively quantified the amount of intraperitoneal fluid as small, moderate or large. Although these last two classifications are often used to predict the haemoperitoneum volume prior to medical treatment of EP, they have never been subjected to a correlation with the volume found at surgery.

Our clinical model for prediction of massive haemoperitoneum may be useful for the medical management of EP. When none of the three criteria is present, medical treatment and follow-up as an outpatient is, to our opinion, possible. Accordingly, the model fits well with well-known countraindications for methotrexate [19] Sowter, 2001; Rozenberg, 2003]. However these contraindications were so far solely based on clinical opinion. Because the absence of all three criteria in the population study was rather uncommon, one may question the usefulness of the model in clinical practice. Our centre has been supporting medical treatment for EP for the last 10 years [10,33] and most patients with obvious criterias for medical treatment were unlikely to be selected in the present study. When used in a more general population of women with EP, the negative predictive value of our model for the diagnosis of significant haemoperitoneum is therefore likely to increase.

Finally, our clinical prediction model should be further tested and may prove useful to monitor patient with methotrexate treatment. Indeed, spontaneous pelvic pain occurs frequently during this treatment. Although in most cases the outcome is spontaneously favourable, this situation does raise concerns and often leads to unnecessary laparoscopy [30,34,35]. Our model may help in the management of these patients: as increasing haemoperitoneum at transvaginal US or a drop in haemoglobin concentration would suggest that laparoscopy should be performed.

## Conclusion

Three criteria may predict massive haemoperitoneum in EP: moderate to severe spontaneous pelvic pain, fluid above the uterine fundus or around the ovary at ultrasound, and low serum haemoglobin concentration. The proposed model accurately predicted significant haemoperitoneum in patients diagnosed to have EP. It could be used to select patients who should not undergo medical treatment. The usefulness of the proposed model should be tested prospectively as part of routine triage of patients with suspected EP in the emergency department.

## Abbreviations

EP: Ectopic pregnancy

US: ultrasound

hCG: human chorionic gonadotropin

## Competing interests

The author(s) declare that they have no competing interests.

## Authors' contributions

All authors read and approved the final manuscript.

AF conceived of the study, and participated in its design and coordination and drafted the manuscript.

AM participated in the design of the study, carried out the review of sonograms, abstracted the data and performed the statistical analysis.

LS helped to perform the statistical analysis, and helped to draft the manuscript.

J-PB participated in the design of the study and helped to carried out the review of sonograms.

YV participated in the design and coordination of the study and helped to draft the manuscript.
